# LESS: Link Estimation with Sparse Sampling in Intertidal WSNs

**DOI:** 10.3390/s18030747

**Published:** 2018-03-01

**Authors:** Xinyan Zhou, Xiaoyu Ji, Yi-chao Chen, Xiaopeng Li, Wenyuan Xu

**Affiliations:** 1 College of Electrical Engineering, Zhejiang University, Hangzhou 310027, China; xinyanzhou@zju.edu.cn (X.Z.); wyxu@zju.edu.cn (W.X.); 2 Department of Computer Science, University of Texas at Austin, Austin, TX 78712, USA; yichao@utexas.edu; 3 Department of Computer Science, University of South Carolina Columbia, Columbia, SC 29208, USA; xl4@email.sc.edu

**Keywords:** link quality reconstruction, topology construction, compressive sensing, wireless sensor networks, environmental monitoring

## Abstract

Deploying wireless sensor networks (WSN) in the intertidal area is an effective approach for environmental monitoring. To sustain reliable data delivery in such a dynamic environment, a link quality estimation mechanism is crucial. However, our observations in two real WSN systems deployed in the intertidal areas reveal that link update in routing protocols often suffers from energy and bandwidth waste due to the frequent link quality measurement and updates. In this paper, we carefully investigate the network dynamics using real-world sensor network data and find it feasible to achieve accurate estimation of link quality using sparse sampling. We design and implement a compressive-sensing-based link quality estimation protocol, LESS, which incorporates both spatial and temporal characteristics of the system to aid the link update in routing protocols. We evaluate LESS in both real WSN systems and a large-scale simulation, and the results show that LESS can reduce energy and bandwidth consumption by up to 50% while still achieving more than 90% link quality estimation accuracy.

## 1. Introduction

The intertidal zone is the area between the high tide line and the low tide line, and long served as a testbed for investigating the impact of environmental conditions such as temperature on the ecology of plants and animals. Due to the unpredictable sea waves, the living conditions of intertidal creatures (e.g., mussels, sea stars, oysters and corals) change significantly. For example, mussels are exposed to the sunlight during the low tide and the temperature of rocks (the habitat for mussels) can go up to 60 ${}^{\circ }$C. When the high tide comes, the sea water cools down the temperature to only 30 ${}^{\circ }$C. Besides temperature, environmental conditions such as humidity, oxygen concentration and pressure also change significantly during a tidal period. Thus, deploying wireless sensor networks in the intertidal areas to grasp the environment changes provides an efficient method for biologists to obtain the first-hand materials.

Deploying a wireless sensor network in the intertidal zone is challenging. In IT-WSNs, packets are expected to be delivered to the sink efficiently with the help of the accurate routing paths. In order to achieve the goal, sensor nodes need to exchange and update the link quality information with neighbors once the link quality changes. However, due to the change of external network conditions in intertidal zones, the network topology may be dynamically changing. For example, wave forces can exceed a category 5 hurricane on an almost daily basis [1] and severe sea waves may impact the communication between nodes and damage the connectivity of the network. Thus, an efficient link quality estimation mechanism is crucial.

Existing link quality estimation methods like 4-bit [2] and EAR [3] perform well when the network is relatively static. However, in IT-WSNs, these methods suffer from a trade-off between performance and energy cost. Specifically, to capture the frequent changes of the network, sensors have to transmit a large amount of beacons which consume a large portion of energy and bandwidth resources. On the other hand, we can limit the energy consumption by decreasing the frequency of beacon updates, yet at the cost of a lower link quality estimation accuracy and, consequently, poorer packet forwarding performance.

In this paper, we strive to mitigate the challenge and manage to strike the balance between accurate link estimation and overwhelming beacon exchanges with sparse sampling. By leveraging the characteristics of link conditions and network system knowledge (e.g., the distribution of sensor nodes and the tide cycle), our approach based on compressive sensing (CS) can obtain high link estimation accuracy while requiring a much fewer amount of link quality measurements and updates.

There are additional challenges to apply CS in link quality estimation. First, CS technique requires the data matrix to be sparse; in reality, however, the original data do not always satisfy this requirement, which means CS cannot be directly used for data reconstruction. Second, the noisy measurement, which is common in real-world sensor networks, may greatly impair the performance of CS [4]. To facilitate the use of CS, we specially design the data matrix, the transform matrix and incorporate the system properties in terms of spatial-temporal correlation.

With the tailored CS design, we propose LESS—a link Quality estimation protocol with fine-grained sparse sampling for IT-WSN systems. The key of LESS is to determine which links among all shall be measured, i.e., the scheduling strategy need to achieve both high estimation accuracy and low communication cost. Furthermore, we find that naive CS scheduling algorithms introduce significant errors in the scheduling process as it fails to distinguish different nodes that play unequal roles in the systems (see Section 4 for details). Therefore, we propose to optimize the scheduling strategies with fine-grained sparse sampling, which further integrates the system knowledge K (e.g., the tide cycle in our systems) as a critical input. Without loss of generality, we design three scheduling algorithms, namely the basic random sampling (RS) without requiring K, K-Spatial and K-Temporal algorithms with the availability of K.

We extensively verify and evaluate the performance of LESS in both systems in George Town and Zhoushan, as well as in a large-scale trace-driven simulation. In summary, the contribution of this paper is as follows:

- We systematically investigate two real-world WSN systems in the intertidal area to reveal the network dynamics. To the best of our knowledge, this is the first measurement work in the real intertidal WSN systems.
- We analyze and verify the feasibility of compressive sensing and design the data and transform matrices to tailor CS for link quality reconstruction.
- We introduce LESS, a link estimation protocol for dynamic networks. We optimize the scheduling strategies and routing path update method with fine-grained system knowledge for various cases.
- We implement and evaluate LESS in the two real-world WSN systems as well as the trace-driven simulations. Exhaustive evaluation results demonstrate that LESS can achieve high link estimation accuracy with limited overhead.

The remainder of the paper is organized as follows. In Section 3, we introduce the motivation of this paper by analyzing the network dynamics and the data property from two intertidal WSN systems. Section 4 introduces the design challenges and solutions in utilizing compressive sensing. In Section 5, we elaborate the design details of our protocol. We introduce the hardware design in Section 6 and Section 7 shows the evaluation results of our protocol. Section 2 discusses the related work and we conclude this paper in Section 8.

## 2. Related Work

The related work can be classified into following categories: link estimation methods, applications of compressive sensing in WSNs and marine sensor networks.

### 2.1. Link Estimation Methods

In the past decades, abundant literature has been proposed for link quality estimation (LQE) to improve routing efficiency. The existing approaches are well surveyed in [5]. Hardware based LQE uses RSS (received signal strength) [6,7,8], LQI (link quality indicator) [9,10,11] and SNR (signal noise ratio) [12,13,14] as indicators while software based LQE exploits metrics such as PRR (packet reception ratio) [15,16,17], ETX (expected transmission count) [18,19,20] and 4-bit [2]. Typical work [2,3,18,21] takes either network layer, link layer or physical layer information as input to get the estimation of link qualities. These methods can well fit in a stable network without environmental change or manual intervention. However, once network condition changes, existing methods such as 4-bit [2] have to replay the estimation process and therefore incur a significant overhead due to information exchange, especially in dynamic networks. Different from the existing work, LESS essentially decreases the information requirements by sparse sampling. As a supplementary technique, LESS can be easily integrated into other methods to enhance their performance.

### 2.2. Applications of Compressive Sensing

Compressive sensing is widely used in WSNs for data collection ([22,23,24]), routing path recovery [25] and topology reconstruction. [26] confirms that compressive sensing prolongs network lifespan significantly with rigorous experiment and theoretical analysis. Liu. [24] utilizes a signal compression algorithm and a reconstruction algorithm based on compressive sampling theory for accurate in-network earthquake timing. CSPR [25] exploits the sparsity that a routing path size is far less than network size and designs a path reconstruction algorithm. NS-RTR [27] utilizes the sparsity of difference between routing path changes and encodes the changes in data packets. Although CSPR [25] and NS-RTR [27] can also achieve topology reconstruction with compressive sensing, the difference from LESS mainly exists in two aspects. First, LESS exploits compressive sensing to recover the basic link quality values instead of data packets. Second, LESS recovers topology based on the recovered link quality values and therefore it can obtain all the potential links besides the routing links (i.e., a complete topology instead of a routing topology). However, the recovered topology for both CSPR [25] and NS-RTR [27] only contains routing links.

### 2.3. Marine WSNs

Marine WSNs are utilized in various applications including water quality monitoring [28], marine creatures monitoring, and ocean current monitoring. The networks structures, hardware design and communication technologies are different across applications. In general, a marine WSN contains a base station and multiple sensor nodes. Nodes can send data to the base station directly or in a multi-hop way. To sustain the requirements of diverse scenarios, sensor nodes may be equipped with temperature sensor, pressure sensor, etc. Many communication technologies are utilized in marine WSNs, e.g., acoustic communication, GSM, and ZigBee. Acoustic communication in Climent et al. [29] is typically used for underwater scenario and cannot be utilized in intertidal areas. SEMAT [30] transmits packets via GSM and sensors are connected with a surface gateway (a buoy). According to the standards of GSM and ZigBee, the battery life of sensors with ZigBee is much longer than that in GSM [31]. Thus, ZigBee is more suitable for wireless sensor networks in intertidal areas. The latest communication technologies, such as LoRa [32] and NB-IoT [33], are also promising to be applied in future marine WSNs.

## 3. Problem Overview and Motivation

In this section, we first investigate the network dynamics and reveal important findings from the measurements of two real-world WSN systems. After that, we look into the data property to verify the feasibility and opportunity of sparse sampling.

### 3.1. Network Dynamics in Intertidal WSN Systems

**Figure 1:**
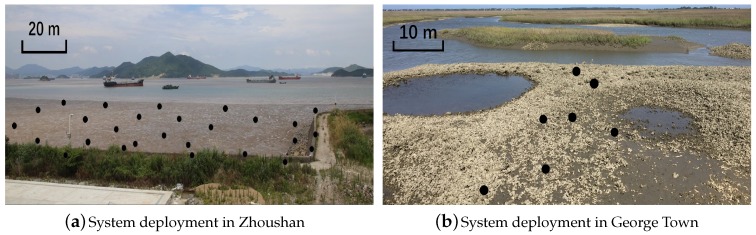
Wireless sensor networks in intertidal zones. We deploy 27 nodes in Zhoushan system and seven in George Town system.

**Figure 2:**
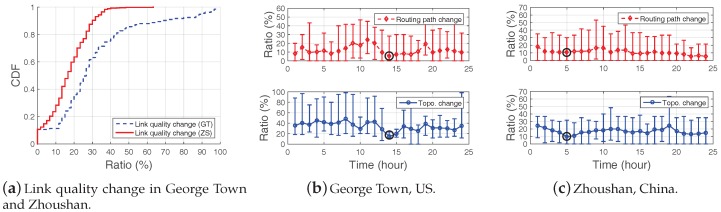
Investigation of the data property in the two systems in George Town (GT) and Zhoushan (ZS): (**a**–**c**) reveal the dynamics of systems, i.e., link quality, routing path, and topology change in 24 h, respectively.

Network dynamics such as link quality, topology and routing path changes are revealed in many WSN systems including GreenOrbs [34], SoilScape [35], OASIS [36], etc. To have a deep understanding of the dynamic characteristics in intertidal WSNs, we deployed two WSN systems in Zhoushan, China (29°56′43″ N, 122°5′10″ E, Figure 1a) and in George Town, US (33°22′37″ N, 79°17′40″ W, Figure 1b) to monitor the intertidal areas. The system details are presented in Section 6. The link qualities in those two systems are heavily impacted by the environments, i.e., the tide movement. The sensors are submerged in the water at a high-tide, and emerged from the water at a low-tide. In addition, sensor nodes are often suddenly lapped by waves. The intertidal environment causes link qualities to change frequently and imposes severe challenges. We quantify the dynamics of the two systems with two 24-h datasets in Figure 2 from three metrics.

- **Link quality change ratio:** Nodes evaluate link quality between neighbors in each cycle, and we compare the link quality change between two continuous cycles. The link quality change ratio is defined as:
  (1) $$Link\;quality\;change\;ratio=\frac{|link\;quality_{ab}\left(t+1\right)-link\;quality_{ab}\left(t\right)|}{link\;quality_{ab}\left(t\right)}*100\%$$
  where $link\;quality_{ab}\left(t\right)$ and $link\;quality_{ab}\left(t+1\right)$ represent the link quality between node *a* and node *b* at cycle *t*, and cycle t+1.
- **Topology change ratio:** During one cycle, any occurrence of new links or disappearance of existing links is defined as a topology change. The topology change ratio is calculated as:
  (2) $$Topology\;change\;ratio=\frac{\#\;of\;cycles\;with\;topology\;changes}{\#\;of\;total\;cycles}*100\%$$
- **Routing path change ratio:** Similarly, any hop change along a routing path from a source to a destination is called a routing path change in one cycle. The routing path change ratio can be represented as:
  (3) $$Routing\;path\;change\;ratio=\frac{\#\;of\;cycles\;with\;routing\;path\;changes}{\#\;of\;total\;cycles}*100\%$$

Figure 2b illustrates the topology and routing path changes ratio in George Town system and Figure 2c demonstrates the case in Zhoushan system, and CTP [37] is utilized as the routing protocol. Figure 2a reveals the link quality change of both George Town system and Zhoushan system. When all sensors nodes are completely submerged under water, no links exist and the change ratio is 0. Therefore, we only take the above-water case into consideration and have the following important findings in Figure 2.

First, the link quality change ratio in the George Town system is higher than that in the Zhoushan system which is shown in Figure 2a. The link quality change ratios of over 60% links in the Zhoushan system are smaller than 20%, while this value is 29.6% in the George Town system. The reason is that the deployment environments in George Town and Zhoushan are different. For example, the tide level in George Town grows with fierce sea waves while the tide level in Zhoushan growing gradually and peacefully. We also find the routing and topology changes in George Town are more remarkable than that in Zhoushan, which implies that link quality change has fundamental impact upon both topology and routing paths.

Second, for both systems, the topology change is more significant than the routing path change. For example, in George Town system, the topology has an average change ratio of 35% while the routing path is only 15% in average. This indicates that topology changes do not necessarily cause routing path changes, which allows us to use partial or sparse topology information yet still obtain accurate routing paths (Section 4).

Third, in both systems, the routing path and topology changes demonstrate time-varying properties, e.g., the change ratios of the 14th hour in Figure 2b and the 5th hour in Figure 2c are relatively small than others. We regard those features as system-specific properties and take them into account as a critical input in the proposed protocol (Section 5).

**Remark:** The change of link quality usually results in that nodes need to update routing paths and broadcast beacons more frequently to grasp the change of the network, which incurs more communication overhead and higher collision probability [5,38,39]. For example, the link quality update mechanism in CTP [37] is called Trickle algorithm [40]. Based on Trickle algorithm, the beaconing interval varies from 64 ms to an hour. That is to say the beaconing interval will increase to one hour if no link quality change occurs, otherwise, nodes should reset the beaconing interval as 64 ms. Thus, nodes send 900 beacons in an hour when link quality changes if they wake up for 1 s per minute, and only 1 beacon if no link changes. Frequent link quality estimation consumes extra energy and declines the system lifespan, and we quantify the energy efficiency after LESS is utilized compared with the one without using LESS in the evaluation section.

### 3.2. Feasibility of Compressive Sensing

After verifying the network dynamics of the two systems and with the above key findings, we now check the prerequisite for compressive sensing—the data should be sparse, or can be transformed to become sparse.

**Figure 3:**
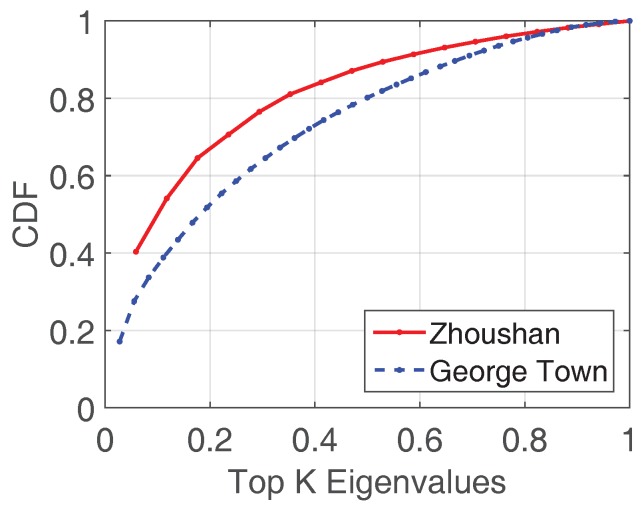
Top k Eigenvalues in George Town and Zhoushan.

There are various methods to estimate the sparsity of a matrix. L0 norm counts the number of zeros in a matrix. However, L0 norm is not convex and does not have a close form [41]. Rank computation [42] is easy but it is sensitive to measurement noise [43]. Therefore, as used in [44], we use the portion of large eigenvalues to estimate the number of principal components which are required to accurately reconstruct the matrix [45]. Figure 3 reveals that the top 50% of the Eigenvalues account for 80% (George Town) and 90% (Zhoushan) “energy”, which indicates the data share low-rank property to some extent. However, the link quality matrix still should be transformed to a sparser form to achieve a better performance.

**Figure 4:**
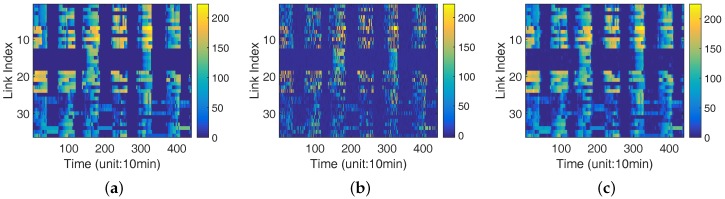
(**a**) Original data matrix (Avg. error = 0); (**b**) processed by standard toolbox in Matlab (Avg. error = 20.69); and (**c**) processed by LESS (Avg. error = 4.89). Heat map for the original (**a**); and recovered (**b**,**c**) data matrices. Larger error is introduced by standard CS than LESS does. (Sampling rate = 0.5, data from George Town system).

To validate the above hypothesis, we compare the performance of data recovery with different algorithms. Figure 4 reveals the heat maps of link quality matrix with different reconstruction algorithms. The values of link quality are within the range of 0 to 255, which are represented from blue to yellow in Figure 4. A higher value of the link quality means a better condition of the link. Figure 4a is a part of link quality matrix we collected in the George Town system, y-axis represents indexes of links while x-axis represents the timeline. Due to the asymmetry of the link quality between two nodes, there are 36 links in the network. Figure 4a is the original data matrix. Figure 4b,c shows the recovered link quality matrices by using standard CS tool in MATLAB and LESS, respectively, under a 50% sampling rate. The estimation errors are 20.69 using CS tool and 4.89 with LESS respectively. The details of LESS is introduced in the next sections.

Note that the estimation error is the average of all the errors for the recovered elements in the data matrix. The result demonstrates that using CS tool straightly does not perform well without elaborate design. The behind reason is that the MATLAB CS tool fails to capture the data property such as the periodic pattern in the two intertidal systems. In Section 4, we talk about how to tailor CS for link quality estimation.

In summary, the intertidal WSN systems demonstrate promising features—highly dynamic, sparse and temporal correlated, which make sparse sampling a good candidate for achieving low communication overhead. We first look into the detailed design challenges and solutions for utilizing compressive sensing in Section 4 and propose the protocol design equipped with compressive sensing in Section 5.

## 4. Utilization of Compressive Sensing

In this section, we first briefly introduce the prime of compressive sensing and then focus on the specific challenges in our systems to utilize compressive sensing for low-cost link quality estimation, i.e., designing the low-rank transform matrix and the measurement matrix.

### 4.1. Prime of Compressive Sensing

Compressive sensing is an effective signal processing technique. By leveraging the presence of structure and redundancy in signals, it reconstructs signals with a sampling rate (SR) which is much lower than the Nyquist rate. Nowadays, it attracts lots of attention in statistics, approximation theory, information theory, and data analytics. It also has many applications in wireless and mobile networks, such as environmental sensing [46], data aggregation [23], and localization [47]. In this paper, we utilize compressive sensing to estimate link quality to decrease communication overhead, which is a first trial.

Consider a time discrete signal *x* with a finite-length *N*, $x\in {I\;R}^{N}$. We measure *x* and observe:

(4) y=Φx+z=ΦΨθ+z

where $y\in {I\;R}^{M}$ are available measurements, Φ is a known M×N measurement matrix (corresponding to the scheduling strategy in Section 5), Ψ is a known N×N representation matrix whose inverse converts *x* to a sparse representation θ, and *z* is an unknown noise term. When θ is sufficiently sparse and Φ obeys a condition known as the Restricted Isometry Property (RIP), x=Ψθ can be reconstructed by solving the convex optimization problem:

(5) $$\underset{\theta }{argmin}{∥y-\Phi \Psi \theta ∥}_{2}^{2}+\lambda {∥\theta ∥}_{1}$$

where the parameter λ can be viewed as a Lagrange Multiplier making a trade-off between the estimation error and the sparsity of the solution. In this paper, we mainly focus on how to tailor compressive sensing for low-cost link quality estimation, i.e., the transform, measurement and constraint matrices. Interested readers can refer to [48] for technical details about the principle of compressive sensing.

### 4.2. Design of the Matrices

We have described the prime of the compressive sensing technology for one-dimensional signals. It can be extended for a two-dimensional matrix and uses Ψ to transform it to a low-rank matrix [44,49,50]. The challenges in our scenario are mainly as follows:

- How to represent the collected data in a proper format to fit for CS.
- What is the best transform matrix Ψ to make the data matrix sparse.
- How to further improve the reconstruction accuracy beyond compressive sensing by taking into account the additional data structure the systems share.

Recall that, in Section 3.2, we mentioned the original data show low-rank but noisy property. Directly using standard CS leads to a larger error, i.e., an absolute error of 20.69 in average, as is shown in Figure 4b, when compared with the original data in Figure 4a. To decrease the errors, we refine the design of the above-mentioned matrices by incorporating the spatial, temporal and system-specific data structures. In Figure 4c, we achieve an absolute error of 4.89 in average, which leads to nearly 90% accuracy (see Section 7 for details). In the following, we elaborate the design of the CS matrices.

**Data Matrix *X*:** In a sensor network with *N* nodes, we represent the link quality between nodes over time as a matrix $X:X\in {I\;R}^{N^{2}\times T}$, where each element X(i,t) represents the link quality of the *i*-th link at the *t*-th time cycle.

**Figure 5:**
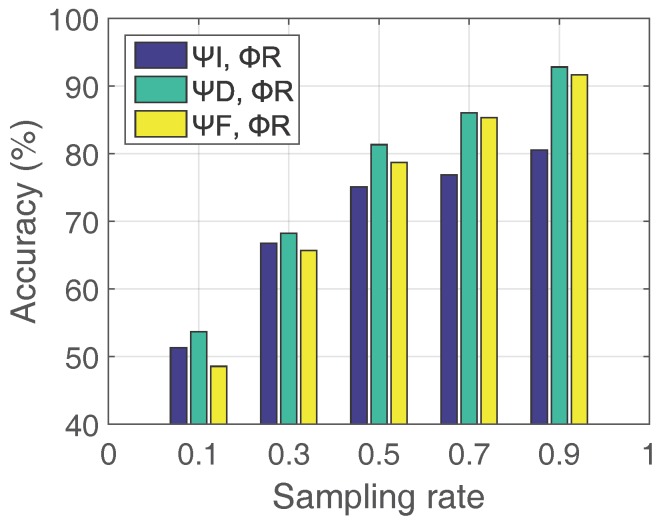
The reconstruction accuracy of link quality matrix with compressive sensing in $\Phi_{R}$ with variant Ψs: identity matrix ($\Psi_{I}$), Fourier transform matrix ($\Psi_{F}$), and discrete cosine transform matrix ($\Psi_{D}$). (George Town data)

**Low-Rank Transform Matrix Ψ:** As we mentioned above, to make *X* a proper low-rank basis, transformations shall be conducted. Generally, the transform matrix Ψ could be Fourier transform, discrete cosine transform, and etc [51,52]. The best Ψ varies for different data matrix *X*. To choose the best transform matrix, we compare three most frequently used transform matrices which are identity matrix ($\Psi_{I}$), Fourier transform matrix ($\Psi_{F}$), and discrete cosine transform matrix ($\Psi_{D}$) in Figure 5. We use a 10-h long data trace collected in George Town system, and reconstruct the link quality matrix with different transform matrices ($\Psi_{I},\Psi_{F},and\;\Psi_{D}$) above with the same measurement matrix: random measurement matrix ($\Phi_{R}$). We calculate the reconstruction accuracy by comparing the reconstructed matrix and the original data matrix, and the result in Figure 5 reveals the discrete cosine transform matrix ($\Psi_{D}$) has better performance than the other two transform matrices for each sampling rate. Therefore, we set Ψ to the discrete cosine transform matrix ($\Psi_{D}$) in our design.

**Measurement Matrix Φ:** There are different strategies to conduct measurement in real-world as shown in Section 5.2. Candes [53] shows that one can stably reconstruct *x* from Equation (5) and the estimated signal $\hat{x}$ obeys:

(6) $$\left|\right|\hat{x}-{x||}_{2}\leq C_{0}k^{-1/2}||x-x_{k}{\left|\right|}_{1}+C_{1}\epsilon$$

where $x_{k}$ is the vector *x* with all but the *k*-largest entries set to zero, $C_{0}$ and $C_{1}$ are constants, and ϵ is the upper bound on the size of the noise *z*. Equation (6) shows that one may observe various magnitude of estimation error when the measurement is noisy.

**Figure 6:**
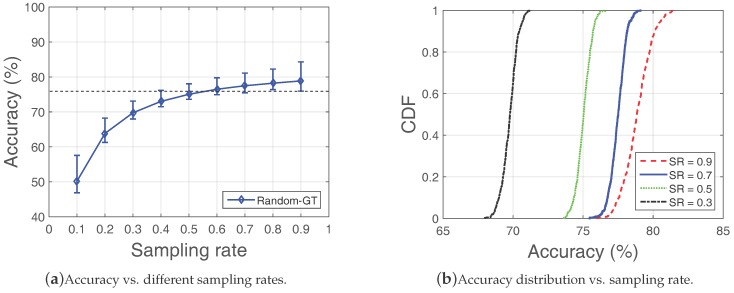
Errors introduced without differentiating the nodes (George Town data).

In Figure 6, we investigate the errors using a random measurement strategy and see the errors in it with the George Town dataset. The results are based on 500 rounds. As shown in Figure 6a, the estimation accuracy for a given sampling rate spans a wide range. Moreover, using a higher sampling rate may not necessarily yield a higher accuracy (e.g., the minimum value with SR=0.9 is even lower than the average value under SR=0.6). Further, we plot the CDF of the accuracy for variant sampling rates in Figure 6b and find that they are evenly distributed. For example, the cumulation of the accuracy increases approximately linearly between 73.4 to 76.4% when the sampling rate is 0.5. This result indicates that estimation errors also distribute evenly. We look into the underlying reason and find there are a few “key nodes” that play critical roles. The errors in random measurements mainly come from the absence of measurements of the “key nodes”. **“Key nodes” are defined as those nodes who has little redundant information**, i.e., the information of those nodes shall be used as much as possible. Table 1 proves the above conjecture. It is clear that under the same sampling rate (SR=0.33), error decreases with the increase of available information from one “key node”. Specifically, the error is 61.8% when the key node is not used and it is only 22.5% if all the information from the node is used.

However, how to decide which node is a “key node” among all the nodes is not that easy. In general, a node with less redundant information is more likely to be a “key node”. The choices of “key nodes” give rise to the importance of Φ, i.e., the scheduling algorithms. A good scheduling algorithm should have more information from the “key nodes” to make the recovery accurate. We introduce the scheduling algorithms which realize Φ in Section 5.2.

**The Regulation Term Design.** From Equation (6), we can see that the performance of compressive sensing technique degrades when data is not sufficiently sparse or the measurement error is large, which are common in real-world data. Therefore, in addition to the low-rank property used by compressive sensing, we introduce and utilize additional data structures to improve the accuracy. Specifically, we consider the spatial and temporal stability in the data and add additional regularization terms to Equation (5):

(7) $$\underset{\theta }{argmin}{∥y-\Phi \Psi \theta ∥}_{2}^{2}+{\lambda ∥\theta ∥}_{1}+\beta {∥P\theta ∥}_{F}^{2}$$

where ${∥\;\cdot \;∥}_{F}$ is the Frobenius norm and *P* is an N×N matrix to capture the temporal stability in θ. An intuitive choice for *P* is the Toeplitz matrix [54] with central diagonal given by ones, and the first upper diagonal given by negative ones, i.e.,

$$P=\left[\begin{array}{cccc} 1 & -1 & 0 & \cdots \\ 0 & 1 & -1 & \ddots \\ 0 & 0 & 1 & \ddots \\ \vdots & \ddots & \ddots & \ddots \end{array}\right]$$

A more sophisticated *P* can be designed based on the system-specific knowledge K (which will be precisely defined in Section 5) to the data. For example, in our application, the tide introduces periodic patterns so the link quality X(i,t) is not only correlated with the last measurement result X(i,t−1) but also with X(i,t−τ), which is the value in the last tide cycle (τ is the tide cycle). Therefore, we can design *P* as:

$$P=\left[\begin{array}{ccccccc} 1 & -\gamma & 0 & \cdots & -(1-\gamma ) & 0 & \cdots \\ 0 & 1 & -\gamma & \cdots & 0 & -(1-\gamma ) & \ddots \\ 0 & 0 & 1 & \cdots & 0 & 0 & \ddots \\ \vdots & \ddots & \ddots & \ddots & \ddots & \ddots & \ddots \end{array}\right]$$

where γ denotes the dependency trade-off among X(i,t−1) and X(i,t−τ) ranging from 0 to 1. In Section 5.2, we elaborate the design of the scheduling algorithms with this consideration. For other systems without periodic patterns, we may utilize other helpful information such as location, geography and etc., to optimize the design of *P*.

## 5. Design of LESS Protocol

Equipped with the tailored CS design, we describe LESS protocol and its components in this section. We first provide an overview of the protocol, and then introduce three sampling algorithms designed for various application scenarios.

### 5.1. Protocol Overview

**Figure 7:**
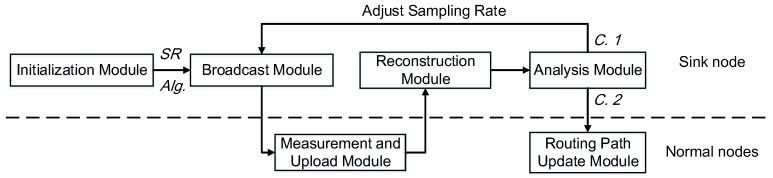
The overview of LESS. The sink node broadcasts the predefined sampling rate and scheduling algorithm, and the normal nodes then measure the link quality and upload the result back to the sink. With the CS technique, the sink node reconstructs the routing paths and analyzes the reconstruction result. From the results, conditions C.1 (current sampling rate is not proper) and/or C.2 (current routing path does not fit) may happen and the protocol goes into corresponding phases.

The protocol is illustrated in Figure 7. We interpret LESS from sink node (which can be connected to a PC and therefore its power and computation capability is not limited) side and normal node side. The sink node is composed of initialization module, broadcast module, reconstruction module and analysis module. The sink node determines the sampling rate (SR) and scheduling algorithm with which the normal nodes perform measurements accordingly. Normal nodes measure link quality and upload the measurement results by embedding them in data packets, e.g., the monitored environment data in our system. The sink node then reconstructs the link quality matrix, thereby calculating proper routing paths.

**Initialization Module:** In this module, the initial sampling rate and scheduling algorithm are determined. The default sampling rate is set to 0.6 and is adjusted based on the measurement results from normal nodes, considering the system requirements, i.e, energy efficiency and accuracy. Besides, the default scheduling algorithm is also chosen in this module based on the availability and property of system knowledge.

**Broadcast Module:** In this module, the sink node broadcasts specified sampling rate (either from the initialization module or from the analysis module) and scheduling algorithm to normal nodes. This module is triggered by the the analysis module if the sampling rate is updated. The broadcast procedure can use protocols such as ADB [55] and BPS [56].

**Reconstruction Module:** The reconstruction module in the sink node computes and reconstructs the link quality matrix *X* from sparsely-sampled measurements. On the sink node side, this module is able to recover the original *X* with the tailored CS with high accuracy. With the recovered *X*, here is the link quality matrix of the network, the sink can decide a routing path for each node with the guidance of typical routing protocols (i.e., Dijkstra shortest path algorithm [57], CTP [37]). Generally, the sink node is more powerful and directly connected with a PC, therefore adding the reconstruction module on sink node will not affect the lifetime of a system due to energy consumption.

**Analysis Module:** This module checks the reconstruction result and determines corresponding strategies under different conditions.

- Condition1(C.1): Update sampling rate. If any of the two conditions are satisfied, sampling rate needs to be increased: (1) over 30% of nodes show deficiency in data collection (PDR decreases up to 20% during the last *10* cycles); and/or (2) did not receive packets from over 10% of nodes during the last *10* cycles. If the following conditions are satisfied, the sampling rate should be decreased: (1) the average PDR increases 20% during the last *10* cycles; and (2) the average PDR stays in a considerable level (over 90%) for over *30* cycles. Note that the lowest sampling rate should equal to the initial sampling rate.
- Condition2(C.2): Update routing path. If the reconstructed link quality matrix produces different and new routing paths, the sink node informs relevant nodes to change their parent nodes.

Occurrence of C.1 and C.2 indicates improper parameters, i.e., sampling rate and routing path setting in LESS. For example, in high-tide period, the sparsity of the link quality matrix is worse than that in low-tide period, therefore a higher sampling rate is demanded.

We then explain LESS from the normal node side.

**Measurement and Upload Module:** With the sampling rate (predefined or updated by the sink node) and the scheduling algorithm, nodes can regulate their behaviors for link quality measurement locally. To alleviate the overhead of transmission, the measured information is attached to normal data packets generated or relayed by the node.

**Routing Path Update Module:** When the normal nodes receive commands for routing path update from the sink, they change their parent nodes immediately. The data format of the command will be discussed in detail later.

Considering the difference of the processing capability between the sink node and normal nodes, most of the calculation work is put at the sink side, and normal nodes only run simple operations such as link quality measurement. In the following, we mainly focus on how to design the scheduling algorithms to achieve high accuracy with low cost.

### 5.2. Scheduling Algorithms

**Problem formulation.** The scheduling procedure can be modeled as a decision problem. Specifically, given a sampling rate *p*, for any node i∈N, a decision matrix $D\in {I\;R}^{N\times T}$ should be determined. D(i,t)=1 specifies that the *i*-th node performs sampling operation (e.g., measurement) at the *t*-th time cycle. Recall that, in Section 4, errors exist in naive random scheduling methods. In our work, we get a precise *D* for various scenarios with the spatial-temporal information of the system.

**System Knowledge K.** K is defined as the special information which can further help decrease sampling rate while maintaining high reconstruction accuracy. For example, link quality values in networks with tree topology are redundant from spatial perspective due to link correlation [58]. As for our intertidal WSN systems, K emphasizes the temporal tide cycle information, which can be obtained form historical data. Intuitively, sampling operations can be performed more wisely with the help of K. In Table 2, we summarize the representation of K in several representative systems.

According to weather and what kind of K is exploited, we propose three classes of scheduling algorithms: the basic random sampling (RS) without K, the K-Spatial sampling (K-S), and the K-Temporal sampling (K-T). We first summarize the notations in Table 3, and then we elaborate introducing the three algorithms in detail.

#### 5.2.1. Random Sampling (RS)

Random sampling assumes that information is randomly distributed in the network, in both spatial and temporal scale. For systems without K, RS provides an intuitive and easy solution for measurements. In RS, link quality information is derived from random nodes at random time instants. Algorithm 1 shows the details of the RS algorithm. RS is implemented locally with a random number generator to produce randomness. As shown in Algorithm 1, if the random number is smaller than the sampling rate, the node performs a measurement and vice versa.

**Algorithm 1** The Random Sampling (RS) Algorithm.**Input:** Number of nodes |N|, sampling rate *s*
**Output:** The decision set *D* for the *N* nodes in time *T*
 1: **for**
i∈N and t∈T
**do** 2:  D(i,t)=Rand()<s
 3: **end for** 4: **return**
*D*

#### 5.2.2. K-Spatial Sampling (K-S)

K-Spatial sampling assumes information is distributed with spatial pattern. For example, in an urban monitoring WSN, nodes are densely deployed in some areas and sparsely deployed in other areas. Thus, we select smaller sampling rate in dense-deployed areas and vice versa. We define the sampling rate in these two ares as $s_{1}$ and $s_{2}$. With a predefined system sampling rate *s*, $s_{1}$, $s_{2}$ and *s*, should satisfy Equation (8).

(8) $$s*|N|=s_{1}*|N_{1}|+s_{2}*\left|N_{2}\right|$$

where $N_{1}$ and $N_{2}$ are the sequences of nodes in dense-deployed areas and sparse-deployed areas, which is so called system knowledge, and $|N_{1}\left|+\right|N_{2}\left|=|N\right|$. According to our analysis above, we also require $s_{1}<s_{2}$. However, there are multiple solution sets $\left(s_{1},s_{2}\right)$ for Equation (8). Here, we introduce $\alpha =s_{2}/s_{1}$ as a controllable parameter which is adaptive for various WSN systems. The best α differs among systems and is related to $|N_{1}\left|/\right|N_{2}|$. In general, we set α=1.5. Together with $s_{1}$ and $s_{2}$, *D* can be derived as shown in Algorithm 2.

**Algorithm 2** The K-Spatial sampling (K-S) Algorithm.**Input:** Number of nodes |N|, $N_{1}$, $N_{2}$, new sampling rate $s_{1},s_{2}$
**Output:** The decision set *D* for the *N* nodes in time *T*
 1: **for**
i∈N and t∈T
**do** 2:  **if**
$i\in N_{1}$
**then** 3:   $D\left(i,t\right)=Rand\left(\right)<s_{1}$ // the dense-deployed case.  4:  **else if**
$i\in N_{2}$
**then** 5:   $D\left(i,t\right)=Rand\left(\right)<s_{2}$ // the sparse-deployed case.  6:  **end if** 7: **end for** 8: **return**
*D*

#### 5.2.3. K-Temporal Sampling (K-T)

K-Temporal sampling assumes information is distributed with temporal pattern. For example, in a WSN deployed in the intertidal zone, link quality changes slightly when nodes are under water (i.e., link quality is always 0) and changes severely when above water. For this case, we can set smaller sampling rate during under-water period and vice versa. For this type of systems, we divide the time period into static phase ($T_{1}$) and dynamic phase ($T_{2}$). We define the sampling rate in these two phases as $s_{3}$ and $s_{4}$, and the predefined system sampling rate *s*, $s_{3}$ and $s_{4}$ should satisfy Equation (9).

(9) $$s*|T|=s_{3}*|T_{1}|+s_{4}*\left|T_{2}\right|$$

Here, $|T_{1}|$ and $|T_{2}|$ are different among systems, and $|T_{1}\left|+\right|T_{2}\left|=|T\right|$. We require $s_{3}<s_{4}$ and define $\beta =s_{4}/s_{3}$ for fitting in different system conditions. We set β=1.5 in general. Together with $s_{3}$ and $s_{4}$, *D* can be derived as shown in Algorithm 3.

**Algorithm 3** The K-Temporal sampling (K-T) Algorithm.**Input:** System running period |T|, $T_{1}$, $T_{2}$, new sampling rate $s_{3},s_{4}$
**Output:** The decision set *D* for the *N* nodes in time *T*
 1: **for**
i∈N and t∈T
**do** 2:  **if**
$t\in T_{1}$
**then** 3:   $D\left(i,t\right)=Rand\left(\right)<s_{3}$ // the stationary phase.  4:  **else if**
$t\in T_{2}$
**then** 5:   $D\left(i,t\right)=Rand\left(\right)<s_{4}$ // the dynamic case.  6:  **end if** 7: **end for** 8: **return**
*D*

**Comparison of the three algorithms.** The three scheduling algorithms are designed for different systems. The random sampling algorithm is of less overhead and suits for general systems, while K-S and K-T are specially designed for systems with spatial and/or temporal characteristics. K-S and K-T require system knowledge in advance, which can be obtained during deployment of systems or from empirical analysis. With the system information, LESS provides better services in both accuracy and efficiency. In Section 7, we give a detailed performance comparison between of three scheduling algorithms.

### 5.3. Routing Path Update

Sink node calculates the best routing path for each node after reconstructing the link quality matrix with sparse sampling. The changes of routing paths are embedded in the update command packets. We design an efficient method to disseminate update commands in the following.

**Figure 8:**
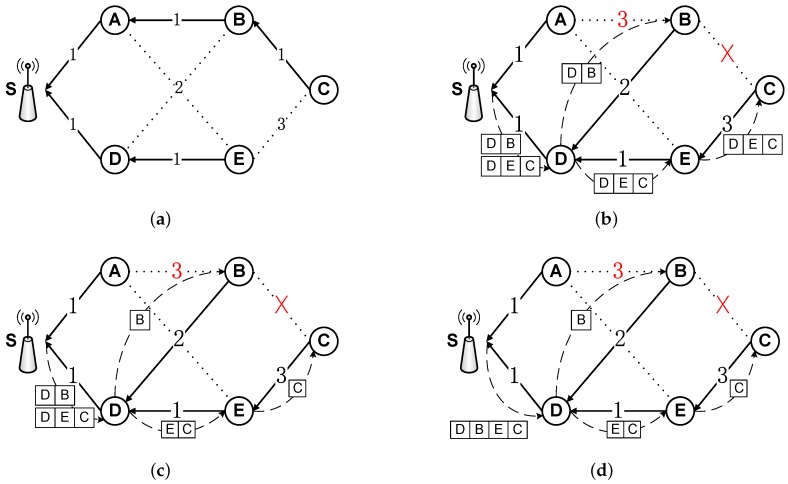
An example of routing path update process with three methods. The directed links represent the information transmission flow. The value on links (including dashed lines) indicate the transmission cost and nodes select the routing paths with the minimum total transmission cost. The dashed lines with arrows are the transmission paths of update packets. (**a**) A simple network topology; (**b**) sending update packets individually to nodeB and nodeC; (**c**) sending update packets with a decreased packet’s length; and (**d**) sending compressed update packets with a decreased packet’s length.

Figure 8a is an illustration with six nodes including a sink. The value labeled on each link represents the transmission cost and the directed arrows indicate data transmission paths. Figure 8b shows two typical reasons of routing path changes: (1) the previous connections are invalid ($link_{BC}$); and (2) an alternative connection with less cost (the cost of $link_{AB}$ is increased from 1 to 3) appears. Note that update commands are necessary for the first case.

Sending update packets with the routing path information node by node is energy consuming, especially in a dynamic network. Figure 8b is a rough dissemination method which carries same information during the forwarding phase with redundancy. To overcome this, we decrease the cost of updating routing path by two steps. (1) Decreasing packet length gradually. Sink node disseminates update packets to target nodes with full routing path information at the beginning. When update packets pass through relay nodes, the information for the relay node is removed from the update packet (Figure 8c). (2) Compressing redundancy. To further improve update efficiency, we compress update packets with identical paths as shown in Figure 8d. Finally, the overhead of delivering routing path update commands can be alleviated.

### 5.4. Discussion

**Compatibility of LESS.** Note that LESS is a supplementary technique and can be easily integrated into existing link quality estimation methods. Considering the fact that existing arts like 4-bit [2] entirely scan the links to obtain an accurate link quality estimation, LESS can be used to decrease the communication overhead while maintaining the accuracy.

**Overhead of LESS.** The overhead of LESS is mainly in three aspects: (1) the delivery of the schedules; (2) the collection of the sampled information; and (3) routing path update. For the first one, the schedules can be delivered using existing dissemination protocols such as [59]. Furthermore, the update process is only invoked when the sampling rate is out of date. As for the second aspect, the link quality information can be embedded into data packets and the overhead can be minimized with coding methods such as [60] and we omit the details in this paper. For the last one, the update command is only sent when a route changes for a node. As mentioned above, the overhead of update commands for routing paths can be resolved by using compression methods shown in Figure 8.

## 6. System Implementation

In this section, we first introduce the hardware design of the sensor node and then describe the architecture of the intertidal WSN.

### 6.1. Sensor Design

**Figure 9:**
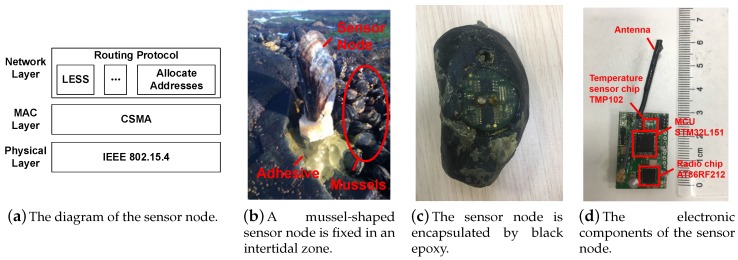
The design of the sensor node: (**a**) the diagram of the sensor node; (**b**) how we deploy sensor nodes in the intertidal area; (**c**) a customized mussel-shape sensor node which is designed for environmental monitoring, and the node is covered by epoxy for waterproof; and (**d**) the electrical components of the sensor node.

In Figure 9a, we show that the MAC protocol for our system is CSMA and PHY layer specification is IEEE 802.15.4. LESS is proposed as a link quality metric estimation method, which can be incorporated into existing routing protocols.

CSMA illustrated in 802.15.4 is easily implemented and widely utilized in environmental monitoring WSNs, including the soil moisture sensing system [35], carbon dioxide monitoring system CitySee [61], and the ocean monitoring system Oceansense [28]. Besides CSMA, TSCH (proposed in 802.15.4e) combines time slotted access and channel hopping and is widely utilized in industrial and vehicular applications. Though TSCH provides energy efficiency services, utilizing TSCH in IT-WSNs in our scenario is challenging. First, TSCH requires tight system synchronization [62]. The IT-WSN can only provide a rough synchronization service with a calibration error around 100 ms due to different clock skew. Intermittent connections caused by tide waves magnify the condition of the time drift among sensors and make time synchronization mechanism deficiency. To implement TSCH in IT-WSN, an elaborately designed time synchronization mechanism is required. On the other hand, TSCH needs re-schedule the time slots when a new node appears, or an existed node disappears, and brings a considerable overhear, especially in IT-WSNs. In IT-WSNs, the changes of available nodes are frequent, thus nodes need to re-schedule TX slots and RX slots frequently and cause extra energy. Based on this, we select CSMA as MAC layer in IT-WSNs. Nevertheless, we wish to investigate the possibility of TSCH in IT-WSNs in future study.

Figure 9b shows a mussel-shaped sensor we designed and deployed near a bed of real mussels. As shown in Figure 9c, the sensor board is encapsulated by black epoxy for waterproof. There are several reasons for us to keep sensor nodes under water:

- Several existing methods keep radio transceivers above water using buoys while keeping sensors underwater. For example, SmartCoast [63] is a wireless sensor network for monitoring water quality utilizing such a method. However, these methods are supposed to operate in lakes, rivers, or estuaries, instead of in intertidal zones where strong waves appear constantly. The strong waves may destroy the cables between the above-water transceivers and the under-water sensors. Besides, buoys will run aground during low-tide periods, and there are high risks that buoys will not float up successfully when high tide comes.
- Intertidal WSNs are designed for monitoring the environmental parameters such as temperature precisely. As a result, sensors should be as close to the creature’s microhabitat as possible, e.g., attached to the rock substrata wherein mussels live. In this condition, connecting sensor nodes to transceivers with wobbly cables will destroy the natural environments of the creatures. Note that to minimize the impact of sensor nodes in our system, we specially designed the sensor nodes as shown in Figure 9c.
- The spots where mussels stay may be far away from the seashore and therefore the water depth is unpredictable. For instance, we observed the water depth in the Zhoushan system can be 5 m. In this case, placing a transmission device on a pole (higher than the high tide level) is impractical.
- Cables may be cut off by creatures such as fiddler crabs in intertidal areas. In addition, human beings are also threats to the cables unless people are forbidden to go into those areas.

Taking above reasons into consideration, we find that encapsulating the sensor nodes entirely and transmitting packets during the low tide period is the best solution for deploying WSNs in intertidal areas. Figure 9d reveals the electronic components of the sensor node. The micro-processor for the sensor node is STM32L151 and its radio chip is AT86RF212 operating at 915 MHz. To save energy, we operate sensor nodes in duty cycle, where they become active for a short period to finish tasks and sleep as much as possible. The cycle period is adjustable and can be controlled by users. For example, a node can be configured to measure physical parameters per 1, 10, or 15 min. The active mode is energy consuming, thus the active time should be short. The switch time for the MCU (STM32L151) from the sleep mode to the active mode is less than 8 μs [64], and transmitting a packet with 128 bytes takes only 4 ms [65] for AT86RF212 (the data rate is 250 kbit/s when the sensor is modulated with QPSK). It takes less than 27 ms [66] for the TMP102 to measure the temperature. Thus, we set the active time as 1 s, which is long enough for the sensor node to deal with measurements and forwarding packets. The theoretical life time of the sensor node is 1.8 years with an 800 mAh lithium battery with a cycle period of 15 min.

### 6.2. Network Architecture

**Figure 10:**
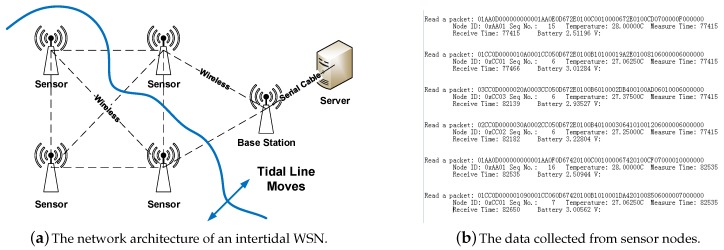
The structure of the IT-WSN and the data process on the server.

Figure 10a is the architecture of an IT-WSN. The IT-WSN is a synchronized multi-hop network which is composed of sensor nodes and a base station. Sensor nodes measure physical parameters and transmit data packets to the base station via wireless communication in multi-hops. The base station is located on the dry land and connect with a server.

### 6.3. Packet Format

Figure 10b reveals the raw data we collected from sensor nodes in hexadecimal, from which we can extract valid information such as node ID, temperature, etc. The collected data are utilized for further study by biologists, which is beyond the scope of this paper.

### 6.4. Network Deployment

We deployed WSN systems in the bay of George Town (South Carolina, USA) and Zhoushan (Zhejiang, China) as shown in Figure 1. The George Town system with seven nodes covers around 3000 ft${}^{2}$ and the Zhoushan system which has 27 nodes covers around 40,000 ft${}^{2}$. The distance between nodes in the George Town system is 15 m on average and the value is 25 m in the Zhoushan system. To have an exhaustive test of the network, we set the cycle period as 1 min.

## 7. Performance Evaluation

In this section, we evaluate LESS in two real intertidal WSN systems and with a large-scale trace-driven simulation. We first compare the performance of LESS with standard interpolation approaches. Then we investigate the reconstruction accuracy of link quality and topology in real systems. We also analyze the energy consumption of LESS with different sampling rates.

### 7.1. Performance Metrics

**Link quality reconstruction accuracy** is calculated by $(1-\frac{\sum \left|lq\_est.-lq\_orig.\right|}{\sum lq\_orig.})\times 100\%$, where lq_est. and lq_orig. are the estimated and original values of link quality. In LESS, we partially sample the link quality in matrix *X*, then reconstruct the entire link quality matrix based on the sampled values. To evaluate the performance of LESS, only unsampled points in *X* are considered in link quality reconstruction accuracy.

**Routing path reconstruction accuracy** is the ratio of correctly reconstructed routing paths given the estimated link quality matrix, i.e., $\frac{\sum correctly\;reconstructed\;routing\;paths}{\sum routing\;paths}\times 100\%$. We obtain the ground truth routing paths with CTP from the original link quality matrix. We then reconstruct routing paths from the link quality matrix with CTP likewise. Finally, we calculate the routing path reconstruction accuracy. The result of the routing path reconstruction accuracy is a direct indicator of the performance of LESS.

**Topology reconstruction accuracy** is defined as the ratio of correctly labeled links in the network, i.e., $\frac{\sum correctly\;labeled\;links}{\sum labeled\;links}\times 100\%$. Each link in the topology is labeled with “0” or “1” to indicate its existence (“1” for yes and “0” for no). A link exists if its link quality value is larger than a threshold (i.e., the noise floor) and vice versa. Topology reflects connectivity of nodes in the network, and provides candidate routes for routing path selection.

The groundtruth of link quality, routing path and topology is from the meta-data of collected packets. The routing protocol used in our systems is CTP.

### 7.2. Evaluation in Real Systems

In this section, we utilize two one-week datasets from the George Town and Zhoushan systems for evaluation.

#### 7.2.1. Comparison with Interpolation

**Figure 11:**
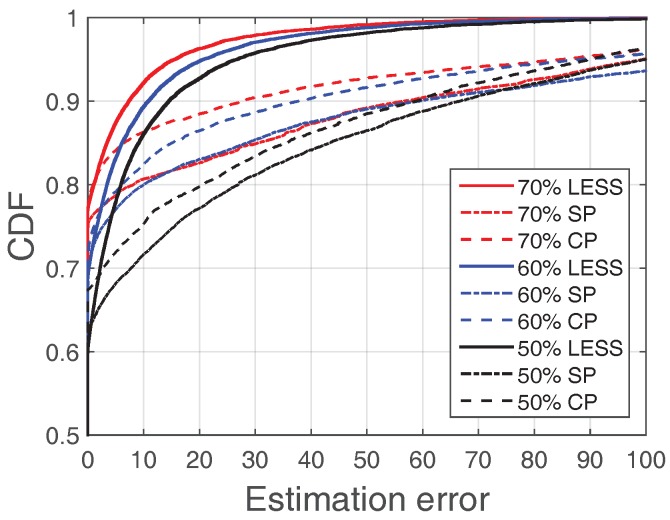
Comparison between LESS-RS and Spline-based approach (SR = 0.5, 0.6, 0.7).

Interpolation is a straightforward method for data reconstruction. We compare the effectiveness of LESS (with random sampling) with two common interpolation methods: cubic spline and cubic polynomial (SP and CP in Figure 11) with different sampling rates based on the experiment results in the Zhoushan system. Figure 11 illustrates the CDF of absolute estimation error values with sampling rates at 50%, 60% and 70%. We find that LESS outperforms SP and CP with all sampling rates. Specifically, when sampling rate is 0.6, 93% of the error is less than 20 in LESS while it is only 80% for SP and 77% for CP. Besides, only 2% errors exceed 50 in LESS while the percentage is around 14% for SP and 12% for CP. LESS has better performance as it involves the spatial-temporal and system knowledge in data processing.

#### 7.2.2. Link Quality Reconstruction

**Figure 12:**
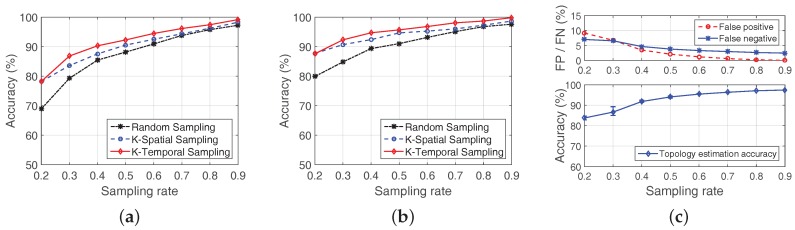
Evaluation of LESS in real trace (Zhoushan system): (**a**) comparison of RS, K-Spatial sampling and K-Temporal sampling in link quality reconstruction accuracy; (**b**) comparison of RS, K-Spatial sampling and K-Temporal sampling in routing path reconstruction accuracy; and (**c**) evaluation of K-Temporal sampling in topology reconstruction accuracy.

We first evaluate LESS with three proposed scheduling algorithms by examining the link quality reconstruction accuracy in Figure 12a with sampling rates from 0.2 to 0.9 in Zhoushan system. For each type of scheduling algorithm, 10 rounds are performed. Generally speaking, the reconstruction accuracy of all scheduling algorithms increases with the growth of sampling rates, especially for sampling rates from 0.2 to 0.5. In the following, we reveal several important findings from the comparison of the three algorithms in terms of link quality reconstruction accuracy.

**Random Sampling vs. K-Spatial Sampling:** RS and K-S demonstrate similar performance when the sampling rate is above 0.7. However, when the sampling rate is less than 0.6, K-S outperforms RS. This is because nodes in Zhoushan system (Figure 1a) are densely deployed, which brings spatial characteristics to the link quality matrix to some extent. In this case, K-S performs better than RS, especially when the sampling rate is small.

**K-Spatial Sampling vs. K-Temporal Sampling:** Comparing the reconstruction accuracy between K-S and K-T in Figure 12a, we observe that K-T behaves better than K-S for all sampling rates. The reason is that K-T benefits from system temporal characteristics, i.e., tide cycle, which is helpful information the intertidal WSNs share.

#### 7.2.3. Routing Path Reconstruction

In addition to the link quality reconstruction, we also verify routing path reconstruction accuracy, which is calculated from the reconstructed link quality matrix. Routing path reconstruction accuracy indicates the capability of LESS in selecting proper next-hop node, although based on link quality reconstruction but is more important than link quality reconstruction in terms of data collection. Figure 12b reveals the relationship between sampling rates and routing path reconstruction accuracy with the three algorithms.

Obviously, the reconstruction accuracy increases with the growth of sampling rates, and reveals the same patterns between RS and K-S and K-T in Figure 12a. It is worth mentioning that, even when the sampling rate is 0.4, the routing path reconstruction accuracy is over 90% in each algorithm and over 80% with a sampling rate of 0.2. Second, routing path reconstruction is better than that of the link quality, especially in low sampling rates. This is because a certain amount of estimation error may not change the final decision in routing path selection. According to Figure 12b, we can draw the conclusion that utilizing compressive sensing in link quality estimation can effectively guide the choice of the next hop with high accuracy.

#### 7.2.4. Topology Reconstruction

We also evaluate the performance of LESS on topology reconstruction using K-Temporal sampling. We test eight traces from different time periods at each sampling rate with K-Temporal sampling in LESS, and the topology recovery accuracy and false positive/negative value are shown in Figure 12c. With the increasing of the sampling rate, the performance of reconstruction accuracy is improved, and over 90% of links are correctly estimated with a sampling rate of 0.4. We look into the false positive/negative statistics and find that both FP and FN are less than 5% when sampling rate is over 0.4. When the sampling rate is extremely low, e.g., 0.1, false positive is over 20% because LESS mistakenly identifies a non-existent link as an existing one. It is also worth mentioning that, even when the sampling rate is 0.2, we can still have a high accuracy, i.e., over 80%, as topology reconstruction requires far fewer data than link quality reconstruction does.

#### 7.2.5. Evaluation of Energy Consumption

**Figure 13:**
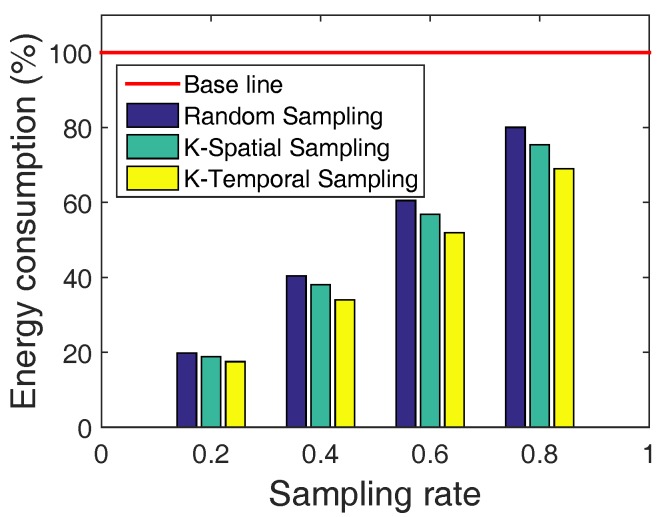
Energy consumption comparisons of link quality estimation between the real trace and LESS (RS, K-S, and K-T) with sampling rates from 0.2 to 0.8.

We also evaluate the energy efficiency of LESS and compare it to the standard CTP protocol. We evaluate CTP and LESS with simulation, and we select the number of beacon packets as an alternative indicator of energy consumption considering the difficulty of reading residual energy in deployed systems. The Trickle algorithm in CTP [37] increases the beacon interval exponentially from 64 ms to 1 h with good links, and resets it to 64 ms when link quality degrades severely. The details of the Trickle algorithm can be found in [40]. We take an 80-h real trace from the George Town system and regard the energy cost of CTP as the baseline, and then compute the energy consumption of LESS with all algorithms. We test all RS, K-S and K-T with different sampling rates from 0.2 to 0.8 and the result is shown in Figure 13. Generally, all three strategies of LESS can decrease energy consumption. Specially, the energy efficiency is nearly linearly related to the sampling rate, e.g., LESS can achieve a 60% energy efficiency with a 0.6 sampling rate. Another conclusion is that both K-S and K-T can further improve the energy efficiency by taking the spatial and temporal knowledge into account. Note that energy consumption of sampling rate updates and link quality information collection is also considered.

#### 7.2.6. Time Complexity Analysis

Both LESS and CTP require nodes to calculate link qualities with neighbors by sending beacons. Taking a network with *n* nodes as an example, every node needs to exchange beacons with a maximum of *n* nodes to maintain a routing table, and the time complexity of CTP is $O(n^{2})$. If the sampling rate of LESS is *s* (s∈(0,1)), n∗s nodes should exchange beacons with a maximum of *n* nodes for link quality reconstruction, and the time complexity of LESS is also $O(n^{2})$.

### 7.3. Large-Scale Trace-Driven Simulations

In this section, we evaluate LESS with a large-scale simulation based on two topologies.

#### 7.3.1. Settings

**Figure 14:**
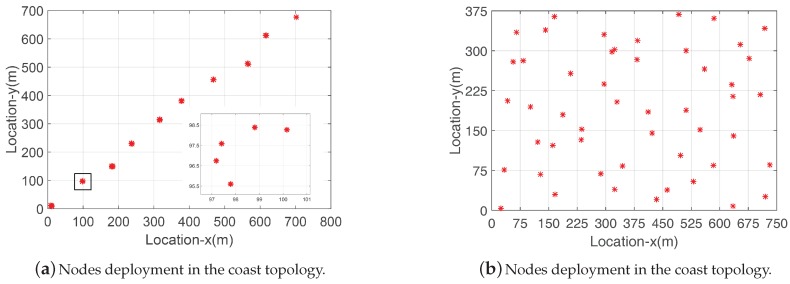
Nodes deployments in the large-scale simulations.

We simulate the performances of LESS in MATLAB in two network topologies—the coast topology and the uniform topology. The coast topology simulates the scenario where sensors are deployed along the coastline in 10 deployment sites. Around 3–8 nodes are randomly deployed at each site, as shown in Figure 14a. The uniform topology in Figure 14b imitates the scenario where the intertidal zone is divided into blocks. A node is randomly deployed in a single block. Each node is assigned with a random altitude value in the network which indicates its deployment height. We exploit real tide datasets which record the continuous changes of water levels to regulate the behaviors of sensor nodes. The tide datasets are from the National Oceanic and Atmospheric Administration [67] and the water level is changing in the range of [0.32 m, 6.602 m]. A node will be submerged when the water level is higher than the altitude of the node.

We deploy 50 nodes in each network, and the maximum communication distance between nodes is set to 150 m. The link qualities between nodes are derived from the signal propagation model with random Gaussian noise. In the simulation, we utilize the K-Temporal sampling algorithm in LESS. We compare the reconstruction accuracies of link quality, routing path and topology in two network topologies with sampling rates from 0.2 to 0.9. We summarize the simulation settings in Table 4.

#### 7.3.2. Results

**Figure 15:**
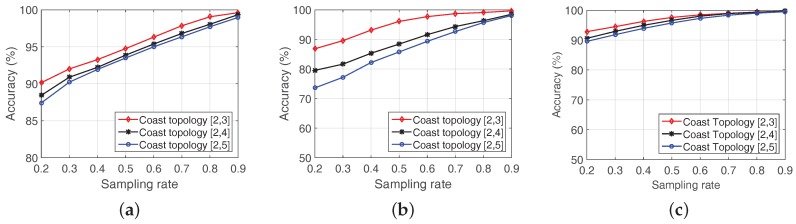
Evaluation of performance in the coast topology: (**a**) link quality reconstruction accuracy in the coast topology; (**b**) routing path reconstruction accuracy in the coast topology; and (**c**) topology reconstruction accuracy in the coast topology.

**Figure 16:**
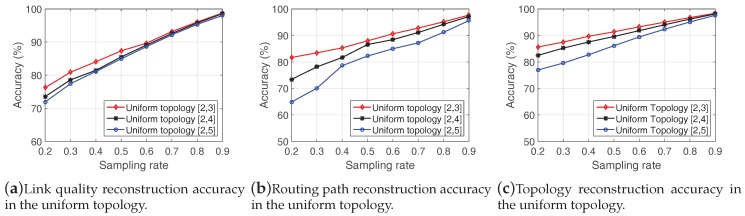
Evaluation of performance in the uniform topology.

We also evaluate the link quality reconstruction, routing path reconstruction and topology reconstruction in the coast and the uniform topology, and the results are shown in Figure 15 and Figure 16.

**Link quality reconstructions in the coast topology & the uniform topology:** Figure 15a shows the estimation accuracy of the coast topology and Figure 16a is the case of the uniform topology. We find that the coast topology demonstrates higher accuracy than the uniform topology at all sampling rates. In the coast topology, nodes are densely deployed in each deployment site while nodes are evenly distributed in the uniform topology (the deployment area is evenly divided into blocks and each block has only one node) as shown in Figure 14. The characteristics of the topology lead to different performance in link quality reconstructions, e.g., nodes in the same deployment site in the coast topology share similar link quality status with each other. As a result, the performance of LESS in the coast topology benefits from the redundancy of link quality.

In addition, we find that parameter of altitude range slightly influences the link quality recovery accuracy with a low sampling rate. For a node, it is more likely to be under water with a lower altitude. When more nodes are under water, the derived matrix is getting sparse as the matrix contains more “0”. The influence of altitude will be elaborated in detail below.

**Routing path reconstruction in the coast topology & the uniform topology:** Unlike for link quality recovery, altitude range reveals significant different performance in routing path recovery in Figure 15b and Figure 16b. Lower altitude range has better performance in both coast topology and uniform topology with similar link quality reconstruction accuracy. Specifically, when the sampling rate is 0.5, the routing path reconstruction accuracy is over 95% with the altitude range of [2,3] while only 88.4% and 85.7% in [2,4] and [2,5] in Figure 15b. The reason is the redundancy in lower altitude range makes the estimation error much evenly distributed in link quality at each point. Thus, the result of the routing path estimation faces less impacts in shortest path routing schemes, i.e., elements in the link quality matrix have similar errors. We also notice the routing path recovery is not as good as in real trace in Figure 12b. The reason is the probable routing paths in real traces and large-scale simulations are in different magnitudes, which leads different performances in the routing path reconstruction. In real-trace experiments, nodes have limited choices of the next hop, and are more likely to select the right node as the next hop. In the large-scale simulation, a node may have over 20 neighbors and link quality reconstruction error may mislead the result of next-hop choices.

**Topology reconstruction in the coast topology & the uniform topology:** As mentioned above, the topology reconstruction error focuses on false positive, i.e., mistaking a non-existent link as an existing one. We analyze topology reconstruction accuracy in Figure 15c and Figure 16c. Like the real-trace evaluation, we set a threshold to eliminate invalid links whose link quality values are less than the threshold.

Altitude range also plays an important role in the topology reconstruction, and lower altitude range has a better performance. The result also matches our deduction that data with redundancy is favorable in data reconstruction for compressive sensing. Besides, the coast topology has a high recovery rate at low sampling rates, e.g., the topology reconstruction accuracy is over 90% when the sampling rate is 0.2 at every altitude range.

#### 7.3.3. The Comparison of Simulation Result and Experiment Result

Comparing the reconstruction accuracy in Figure 12a, Figure 15a and Figure 16a, we find that LESS performs the best in the simulation of coast topology, and LESS has better performance in Zhoushan system than that in the simulation of uniform topology. In the Zhoushan system, we deploy sensor nodes near the coast line, and several nodes are deployed far from the seashore. Thus, the sparsity of the link quality matrix in Zhoushan system is between the coast topology and the uniform topology. This is also the reason why LESS performs the best in the coast topology.

## 8. Conclusions

This paper proposes LESS, an efficient link reconstruction protocol by utilizing sparse sampling for intertidal WSNs. LESS designs the CS matrices which take advantages of the spatial-temporal property as well as the system knowledge as input to optimize the link quality reconstruction accuracy with low overhead. Evaluation is conducted in both real systems and a large-scale trace-driven simulation. The evaluation results show that LESS can reduce the bandwidth and energy consumption by 50% while achieving 90% link quality estimation accuracy.

## Figures and Tables

**Table 1:** Errors vs. key node information percentage (SR=0.33).

Key Node Info.	0%	25%	50%	75%	100%
Average Error	61.8%	49.5%	46.1%	28.6%	22.5%

**Table 2:** The system knowledge K in different WSNs.

WSNs	The System Knowledge K
Intertidal WSN	Tide schedule, the location of nodes
GreenOrbs [34]	Weather, the location of nodes
SoilScape [35]	Weather, moisture, depth of nodes
OASIS [36]	Temperature, the location of nodes

**Table 3:** Parameter notations in LESS.

i, N	N is the set of nodes, and i∈N.
t, T	T is the running period, and t∈T.
K	The system knowledge. In the intertidal zone, K is reflected in β.
*s*	Sampling rate of the system
α	User-defined weight coefficient of sampling rates in systems with spatial characteristics. $\alpha =s_{2}/s_{1}$
β	User-defined weight coefficient of sampling rates in systems with temporal characteristics. $\beta =s_{4}/s_{3}$.
D(i,t)	*D* is the decision matrix; node *i* will be sampled at time *t* if D(i,t)=1.

**Table 4:** The values of simulation parameters.

Topology	Coast topology and uniform topology
Network size	50 nodes in each network
Communication range	150 m
Nodes altitude distribution	[2,3], [2,4] or [2,5] (unit: meter)
Sampling rate	0.2, 0.3, 0.4, 0.5, 0.6, 0.7, 0.8, 0.9
